# Ferroptosis and Autoimmune Diseases

**DOI:** 10.3389/fimmu.2022.916664

**Published:** 2022-06-03

**Authors:** Benjamin Lai, Chien-Hsiang Wu, Chao-Yi Wu, Shue-Fen Luo, Jenn-Haung Lai

**Affiliations:** ^1^ Department of Internal Medicine, Chang Gung Memorial Hospital, Taoyuan, Taiwan; ^2^ Division of Allergy, Immunology, and Rheumatology, Department of Internal Medicine, Chang Gung Memorial Hospital, Taoyuan, Taiwan; ^3^ Division of Allergy, Asthma, and Rheumatology, Department of Pediatrics, Chang Gung Memorial Hospital, Taoyuan, Taiwan; ^4^ College of Medicine, Chang Gung University, Taoyuan, Taiwan; ^5^ Graduate Institute of Medical Science, National Defense Medical Center, Taipei, Taiwan

**Keywords:** ferroptosis, systemic lupus erythematosus, rheumatoid arthritis, inflammatory bowel diseases, inflammation

## Abstract

Adequate control of autoimmune diseases with an unclear etiology resulting from autoreactivation of the immune system remains a major challenge. One of the factors that trigger autoimmunity is the abnormal induction of cell death and the inadequate clearance of dead cells that leads to the exposure or release of intracellular contents that activate the immune system. Different from other cell death subtypes, such as apoptosis, necroptosis, autophagy, and pyroptosis, ferroptosis has a unique association with the cellular iron load (but not the loads of other metals) and preserves its distinguishable morphological, biological, and genetic features. This review addresses how ferroptosis is initiated and how it contributes to the pathogenesis of autoimmune diseases, including systemic lupus erythematosus, rheumatoid arthritis, and inflammatory bowel diseases. The mechanisms responsible for ferroptosis-associated events are discussed. We also cover the perspective of targeting ferroptosis as a potential therapeutic for patients with autoimmune diseases. Collectively, this review provides up-to-date knowledge regarding how ferroptosis occurs and its significance in autoimmune diseases.

## Introduction

Autoimmune diseases are a group of complicated diseases with unknown etiologies. Different autoimmune diseases may share certain similarities in their clinical presentations and yet preserve unique characteristics in each individual autoimmune disease. For example, patients with rheumatoid arthritis (RA) suffer mainly from polyarthritis involving the hand joints, and the extra-articular major organ like kidney is less commonly involved. Different from RA, patients with systemic lupus erythematosus (SLE) may have multiple organ involvement due to overproduction of a variety of autoantibodies and the deposition of antibody-antigen immune complexes in various organs, such as the kidney, causing organ damage. In comparison, although oligoarthritis can occur in certain populations of patients with inflammatory bowel disease (IBD), the major presentation of IBD is chronic intestinal inflammation. Extensive studies have been conducted to identify the factors causing immune activation in autoimmune diseases. One of these directions has focused on investigating the occurrence of abnormal or increased cell death and the inadequate clearance of dead cells that leads to the release of the intracellular contents from the dead cells to trigger inflammatory reactions.

While screening for antitumor compounds, the group led by Stockwell BR synthesized and identified two major compound types displaying synthetic lethality with oncogenic RAS in cancer cell lines. One of these compounds is erastin, and the other is RAS synthetic lethal 3 (RSL3) and RSL5, which facilitate iron-dependent, nonapoptotic cell death in cancer cells expressing an oncogenic isoform of RAS (1, 2). The authors observed that cells transformed with oncogenic RAS have an increased iron load compared with their untransformed counterparts by upregulating transferrin receptor 1 and downregulating ferritin heavy chain 1 and ferritin light chain (2). Erastin and RSLs do not target RAS but rather bind to voltage-dependent anion channels to induce RAS-RAF-MEK-dependent oxidation and nonapoptotic cell death (3). These studies raised an interesting observation that links nonapoptotic cell death with cellular iron load.

Ferroptosis was first proposed by Dixon et al. based on the observation that cell death induced by erastin or RSL showed an increase in cytosolic, and lipid reactive oxygen species (ROS) and cell death can be suppressed by cotreatment with the iron chelator deferoxamine (4). Coincubation with different exogenous sources of iron, but not other divalent metal ions, such as Cu^2+^, Mn^2+^, Ni^2+^, and Co^2+^, potentiated erastin-induced cell death (4). Several features of ferroptosis, including loss of membrane integrity, increased membrane density, shrinking mitochondria and rupture of the outer mitochondrial membrane with morphologically normal nuclei distinguish this death subtype from other cell death subtypes, such as apoptosis, necrosis and autophagy (4–7). Phenotypically, ferroptosis shares a certain similarity with H_2_O_2_-induced necrosis; however, the ATP pool that is depleted in H_2_O_2_-induced necrosis is not affected in ferroptosis (4). Different organelles, such as lysosomes and the endoplasmic reticulum (ER), are involved in the processes of ferroptosis (8). The authors analyzed a shRNA library targeting mainly mitochondrial genes and observed that erastin-induced ferroptosis was regulated by a subset of genes, such as ribosomal protein L8, iron responsive element binding protein 2, *ATP5G3*, TPR repeat protein 35, citrate synthase and acyl-CoA synthetase family member 2, which are different from those involved in staurosporine-induced apoptosis (4). The studies thus provide strong evidence that an iron-dependent accumulation of ROS contributes to ferroptosis. Following these early studies, subsequent works revealed that ferroptosis occurs in many clinical disorders, such as kidney disease, cardiomyopathy, atherosclerosis, neurodegenerative disorders, ischemia/reperfusion (I/R) injury, cancers and immune-mediated diseases, such as diabetes, multiple sclerosis, and asthma (9, 10). In comparison, given that most of the published studies have focused on investigating the roles of ferroptosis in cancer (11), knowledge about the involvement and mechanisms of ferroptosis in autoimmune diseases remains largely unclear.

To discuss how ferroptosis is involved in the pathogenesis of autoimmune diseases, some prototypic autoimmune diseases were chosen based upon the accessible messages from published work. Accordingly, several autoimmune disorders, such as SLE, RA, and IBD, were chosen as examples for a discussion of ferroptosis. Importantly, several interesting studies recently reported that ferroptosis contributes to the pathogenesis of these three distinctive autoimmune diseases that do share some similarities. In addition to ferroptosis, other subtypes of cell death such as apoptosis, necroptosis and pyroptosis can also be detected in immune or non-immune cells in patients with SLE, RA and IBD (12–18). Furthermore, in addition to that in SLE, RA and IBD, ferroptosis also play roles in the pathogenesis of multiple sclerosis, an autoimmune neurological disorder, with mechanisms involving the reduced expression of Gpx4 mRNA in the gray matter of multiple sclerosis patients and decreased Gpx4 protein levels in experimental autoimmune encephalomyelitis mice (19). This review specifically addresses how ferroptosis occurs and the factors that trigger ferroptosis as well as the underlying mechanisms in patients with SLE, RA and IBD. Furthermore, we will discuss the perspective of targeting ferroptosis to achieve better therapeutic control of autoimmune diseases.

### Distinguishing Features of Different Cell Death Subtypes

Apoptosis is a noninflammatory form of programmed cell death mediated by the activation of caspases that occurs in a well-organized manner (**Table 1**). Apoptosis is generally considered a mechanism that occurs under physiological conditions (20, 21). Apoptosis can be executed by sensing intrinsic signals such as cell stress or by sensing extrinsic signals delivered from outside cells as the commonly recognized intrinsic pathway and extrinsic pathway, respectively. Activation of the intrinsic pathway depends on the release of mitochondrial proteins from the mitochondria, whereas activation of the extrinsic pathway is mediated by extracellular ligands that bind to death receptors on the cell surface; caspases, such as caspases 3, 8 and 9, are involved in apoptotic pathways (22). In addition to caspases, B-cell lymphoma-2 (Bcl-2) family members, death receptors, and members of the tumor necrosis factor (TNF) receptor superfamily are individually involved in the intrinsic and extrinsic apoptotic pathways (23). Aside from the changes in mitochondria (24), the characteristic features of apoptosis are membrane blebbing, chromatin condensation and margination, and exposure of membrane phosphatidylserine that induces phagocytosis of apoptotic cells by macrophages (25, 26). Importantly, the intact plasma membrane during apoptosis prevents the release of intracellular content, a process that can potentially trigger overwhelming immune responses. In comparison, a unique feature of necroptosis is swelling of the cytoplasmic organelles, dilated perinuclear space and permeabilization and rupture of the plasma membrane, suggesting the morphological features of necrosis (27–29). In addition, under electron microscopic examination, the electron density of the nucleus is maintained in the early phase and then the nucleus progresses to show sickle-like morphology in the late phase due to compression of the inner nuclear membrane by the dilated perinuclear space (27). The mechanisms of necroptosis involve the formation of a signaling platform named necrosome, the core machinery of the necroptosis death complex that consists of mainly receptor-interacting protein kinases (RIPK1 and RIPK3) and pseudokinase mixed lineage kinase domain-like (MLKL) (29–31). The resulting rupture of the plasma membrane extensively involves the activation of different kinases, such as MLKL, IkappaB kinase (IKK)ϵ, and TANK-binding kinase 1 (TBK1), as well as participation by the ubiquitination process. Although commonly recognized as a host defense mechanism against viral infection, recent studies have suggested that unsuccessful caspase-mediated cleavage of RIPK1 may lead to autoinflammatory syndrome (32, 33).

**Table 1:** Distinguished features of different subtypes of cell death*.

	Apoptosis (22-26)**	Necroptosis (27-33)	Pyroptosis (34-41)	Ferroptosis (4, 8, 27, 45-47)
Membrane features	Membrane blebbing; exposure of membrane phosphatidylserine	Membrane permeabilization and disruption	Formation of membrane pores and membrane rupture	Rupture of plasma membrane (due to accumulation of high lipid peroxides)
Nuclear characteristics	Nuclear shrink, nuclear fragmentation, chromatin condensation and margination	Maintaining nuclear electron density in early phase and becoming sickle-like morphology of nucleus due to compression of the inner nuclear membrane by the dilated perinuclear space (under EM)	Peculiar form of chromatin condensation and intact nuclei	No nuclear morphological changes but with lucent nucleus under EM and yet with oxidative damage of nuclear DNA
Organelle morphology	Swollen mitochondria, mitochondrial outer membrane permeabilization	Cytoplasmic swelling, opening of the mitochondrial permeability transition pores	Intact and swollen mitochondria with reduced matrix density and collapsed cristae (under EM)	Swollen mitochondria, increase in mitochondrial membrane density, disappearance of mitochondrial cristae
Biochemical determinants	Caspases (2, 3, 6-10), Bcl-2 family members, death receptors, TNF receptor superfamily members	RIPK1, RIPK3, MLKL, IKKϵ, TBK1	Caspases (1/4/5 in human; 11 in mice), IL-1β, IL-18, GSDMD	Iron load, Gpx4, ACSL4, POR, CYB5R1, lipid peroxidation

* There are interactions or overlapping features among different subtypes of cell death and thus the listed features are not necessarily excluding other uncovered features. In addition, different death triggers happening in different tissue cells may also cause variations.

**, references.

Bcl-2, B-cell lymphoma 2; TNF, tumor necrosis factor; RIPK, receptor-interacting serine/threonine-protein kinase; MLKL, Mixed lineage kinase domain-like; IKK, I-kappaB-alpha kinase; TBK1, TANK-binding kinase 1; IL-1, interleukin-1; GSDMD, gasdermin D; ACSL4, acyl-CoA synthetase long chain family member 4; POR, NADPH-cytochrome P450 reductase; CYB5R1, NADH-cytochrome b5 reductase; EM, electron microscope.

The formation of plasma membrane pores resulting in dysfunctional ion fluxes, intact but swollen mitochondria with reduced matrix density and collapsed cristae, a peculiar form of chromatin condensation and intact nuclei, and cell lysis are characteristic features of pyroptosis (34–38). Pyroptosis is a highly inflammatory form of cell death that is commonly observed with infection by intracellular pathogens and functions as part of the antimicrobial immune responses (35, 39). The formation of a large supramolecular complex called the inflammasome in response to intracellular danger signals is a hallmark of pyroptosis and mitochondria is a major organ regulating pyroptosis and metabolism (40). In the inflammasome, the activation signal triggers caspase signaling pathways (involving caspase-1/4/5 in humans and caspase-11 in mice; these caspases are different from those involved in apoptosis) and results in the cleavage of pro-interleukin (IL)-1β, pro-IL-18 and gasdermin D (GSDMD), a pore-forming protein (21, 37). The formation of pores then disrupts the cell membrane and releases intracellular molecules such as high-mobility group box 1 (HMGB1), ATP, DNA, and proinflammatory cytokines, which then perpetuate the inflammatory responses and cause tissue injury (41). Given that the different subtypes of cell death preserve their individual unique features and the underlying mechanisms and involved molecules vary, crosstalk or linkages among the different cell death pathways exist. One example is that RIPK1 and caspase-8 regulate the crosstalk between apoptosis, necroptosis, and pyroptosis (42). The interaction between autophagy and apoptosis involving a complex array of biomarkers to coordinate and regulate cell survival or death was also recently addressed in cancers (43).

In addition to the distinguishable morphological changes in ferroptotic cells, one unique feature of ferroptosis is that it does not involve death receptors or sensers to trigger the ferroptotic processes (21). The signaling pathways involved in apoptosis and other cell death subtypes, including cytochrome c release, caspase activation, and activation of receptor-interacting protein 1 and MLKL, do not necessarily participate in ferroptosis (44). Another striking characteristic of ferroptosis is the involvement of iron metabolism dysregulation that results in the accumulation of high amounts of lipid peroxides in cellular membranes (4). Following events such as lipid peroxidation and formation and the accumulation of cytotoxic adducts, the plasma membrane is disrupted, damage-associated molecular patterns like HMGB1 is released and activated and cellular function is damaged (45, 46). The morphological changes of ferroptosis include intact but lucent nucleus under electron microscopy and changes of mitochondria, including swollen mitochondria, increase in mitochondrial membrane density, and disappearance of mitochondrial cristae (8, 27). Although studies suggested that the oxidoreductases, including NADPH-cytochrome P450 reductase (POR) and NADH-cytochrome b5 reductase (CYB5R1) may be responsible for causing membrane damage in ferroptosis (47), it remains unclear whether any specific death-inducing proteins or protein complexes are responsible for causing the accumulation of ROS and the execution of ferroptosis. A detailed review of the different subtypes of cell death in endocrine diseases has been reported in a sophisticated manner by Tonnus et al. (21).

### Dysregulated Iron Metabolism and Oxidative Responses in Ferroptosis

Iron is critical for the survival and reproduction of all mammals. Under physiological conditions, most iron is contained in red blood cells or forms a transferrin complex (Tf-Fe^3+^) that circulates in the bloodstream to supply oxygen to the tissue cells from red blood cells (48). Aging red blood cells are undergoing phagocytosis mainly by macrophages, and hemoglobin-derived iron must be recycled back to the circulation for utilization by erythropoietic cells for hemoglobin synthesis. Both ferrous (Fe^2+^) and ferric (Fe^3+^) iron integrated within iron-sulfur (Fe-S) clusters, hemoproteins and ribonucleotide reductases mediate various biological functions, such as maintenance of the electron transport chain in the mitochondria, cellular respiration, energy production, and DNA replication and repair (49). Furthermore, iron is a crucial regulator in resuming the functions of enzymes such as catalases, peroxidases, lipoxygenases, cyclooxygenases, and nitric oxide synthases (11). Given that the bioavailability of iron is low under physiological conditions and that iron is important for commensal and pathogenic bacteria to grow, the host coordinates immune mechanisms to limit the accessibility of iron to bacterial invaders and fight against bacterial infection (50).

Iron as an electron carrier also functions as a redox catalyst in the Fenton and Haber-Weiss reactions, resulting in the generation of ROS (51). In response to oxidative stress, various forms of ROS, including superoxide radicals, hydrogen peroxide, hydroperoxyl radicals, hydroxyl radicals and lipid-associated ROS (such as hydroperoxides, peroxyl radicals and alkoxyl radicals), and iron or iron-containing complexes are generated and participate in the formation or decomposition of ROS (52, 53). To counteract oxidative stress, iron must lose electrons to undergo the necessary chemical reactions. Thus, because iron is involved in various reactions to maintain body homeostasis, the mechanisms of maintaining cellular iron levels are critical, and excess intracellular iron triggered by exaggerated oxidative stress will be toxic and cause damage to proteins, DNA and the plasma membrane (49, 51, 54). As one of the recognized effects, the dysregulation of iron metabolism may lead to the iron-dependent accumulation of lethal lipid peroxides in the phospholipid bilayer of the plasma membrane, a mechanism contributing to ferroptosis (4).

**Figure 1:**
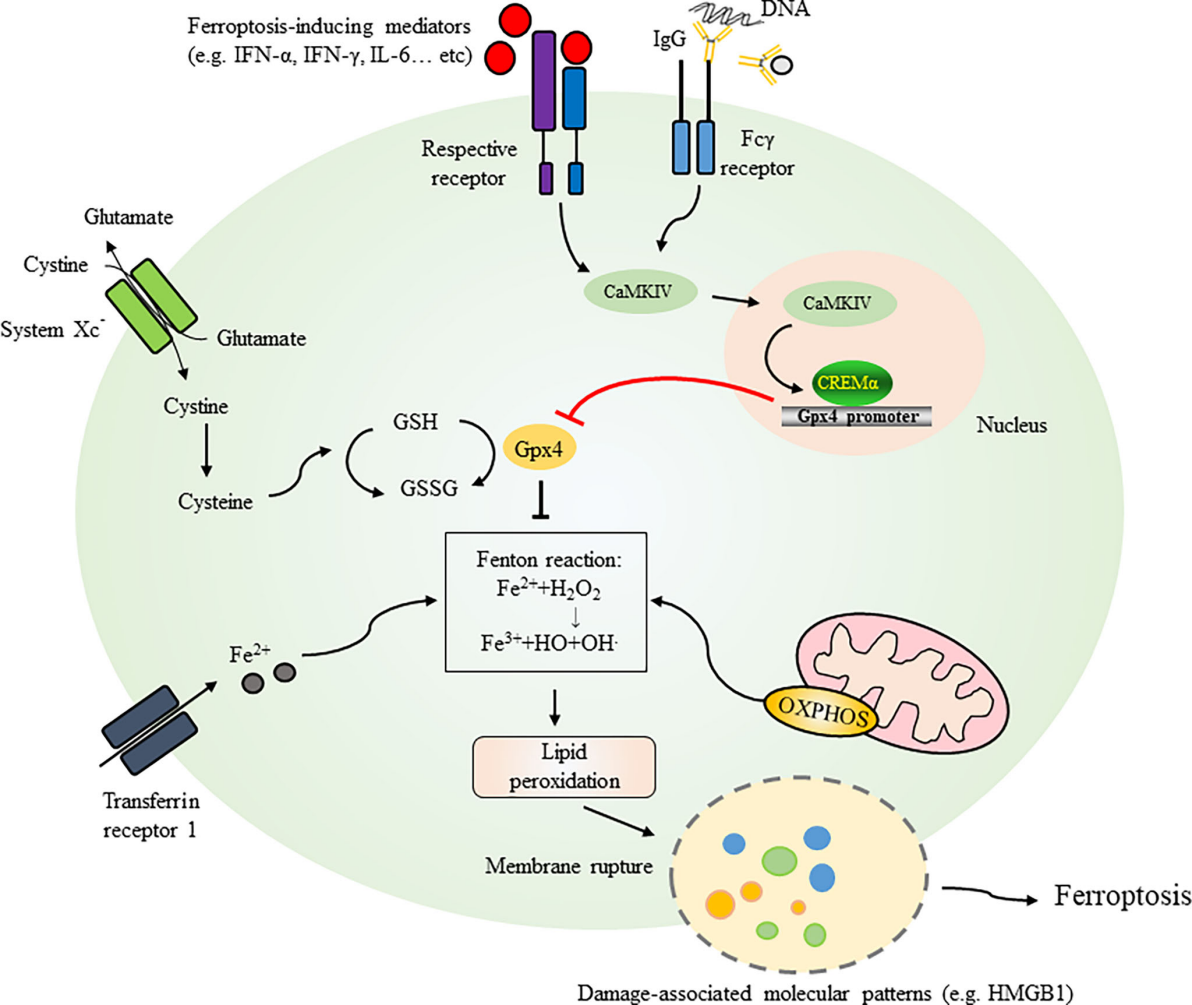
Initiation and mechanisms regulating ferroptosis. Several key factors, including cystine uptake, levels of GSH, and activities of Gpx4, act as crucial regulators counteracting iron-dependent generation of lipid peroxidation products and preventing ferroptosis and plasma membrane damage. One molecule of cystine across plasma membrane with an exchange of one molecule of glutamate through cystine-glutamate antiporter (System Xc^-)^. Inside the cell, cystine is reduced to cysteine for GSH synthesis. GSH is easily oxidized to the disulfide dimer GSSG (oxidized form of glutathione). Extracellular iron can be transported to intracellular through transferring protein 1. Iron as an electron carrier also functions as a redox catalyst in the Fenton and Haber-Weiss reactions resulting in the generation of ROS. Mitochondria are critical organelle containing ferroptosis-related genes and regulate generation of lipid peroxides through the electron-transporting chain. Ferroptotic pathway can be initiated by activation of cytokines such as IFN-α, IFN-γ and IL-6 or serum IgG (e.g. in SLE patients) leading to the activation of CaMKIV/CREMα axis as an example demonstrated in SLE. Binding of CREMα to Gpx4 promoter resulted in suppression of Gpx4 expression which regulates biosynthesis of GSH. Gpx4 also catalyzes the reduction of oxidized biolipids, that converts toxic lipid hydroperoxides into non-toxic lipid alcohols through its cofactor GSH. Dysregulation of these factors leads to ferroptosis with release of damage-associated molecular patterns like HMGB1 and membrane rupture. GSH, glutathione; GPX4, glutathione peroxidase 4; CaMKIV, Ca^2+^/calmodulin–dependent kinase IV; CREMα, cAMP-responsive element modulator α; IFN-α, interferon-alpha; IFN-γ, interferon-gamma; IL-6, interleukin-6; HMGB1, high mobility group box 1; OXPHOS, oxidative phosphorylation.

The cellular homeostatic mechanisms preventing damage to the cell membrane from lipid peroxides occur *via* glutathione peroxidase 4 (Gpx4), a selenoenzyme responsible for catalyzing the reduction of oxidized biolipids that converts toxic lipid hydroperoxides into nontoxic lipid alcohols through its cofactor glutathione (GSH) (55–58) (**Figure 1**). Inside the cell, cystine is reduced to cysteine and then cysteine is used by the enzymes c-glutamyl cysteine synthetase and glutathione synthetase for the synthesis of GSH (59, 60). One molecule of cystine, a predominant extracellular form of cysteine, crosses the plasma membrane in exchange for one molecule of glutamate through a cystine-glutamate antiporter (system Xc^-^) (61). This cystine/glutamate transporter system also plays critical roles in maintaining intracellular glutathione levels and the redox balance in the extracellular space. The expression of system Xc^-^ is regulated by the nrf2-keap1 pathway (nrf2, nuclear factor E2-related factor-Kelch-like ECH-associated protein 1) (62, 63) and is increased under oxidative stress (64). Given that the cellular cystine/cysteine/GSH/Gpx4 axis is an important pathway to protect cells from undergoing ferroptosis (9), any events triggering oxidative stress or generating inhibitory events on the cystine-glutamate antiporter (system Xc^-^) by increasing the level of glutamate or an exogenous stimulant, such as sulfasalazine, can deplete intracellular GSH, reduce Gpx4 substrate availability and disrupt the axis (4). Collectively, several key factors, including cystine uptake, GSH level, and Gpx4 activity, act as upstream regulators that counteract the iron-dependent generation of lipid peroxidation products and prevent ferroptosis and plasma membrane damage (53). Studies have shown that Gpx4-knockout mice die at the same embryonic stage, and cells from mice with inducible Gpx4 inactivation exhibit 12/15-lipoxygenase-derived lipid peroxidation and apoptosis-inducing factor (AIF)-mediated cell death (58). In addition to showing that neuron-specific Gpx4 depletion results in a neurodegenerative phenotype in mice (58), inducible Gpx4 depletion manifests with a very large amount of renal tubular epithelial cell death that leads to acute renal failure and early death in mice (5).

While inhibition of system Xc^−^-mediated cystine uptake is sufficient for erastin-induced ferroptosis, it may not be necessary to execute ferroptosis given that RSL3-induced ferroptosis shares similar patterns with erastin-induced ferroptosis, which does not inhibit cystine uptake. How the increased iron load induces ferroptosis remains unclear because H_2_O_2_ treatment also results in iron-catalyzed ROS production; however, H_2_O_2_-induced cell death patterns are different from ferroptosis (4). Indeed, up-to-date research has still not disclosed the signals that ignite message transmission leading to ferroptosis (65). In addition, it remains unclear whether the phenotypic changes seen in ferroptotic cells are a consequence of the disruption of the plasma membrane due to extensive lipid peroxidation or a consequence of downstream signaling-mediated events. More comprehensive reviews about the mechanisms and processes involved in ferroptosis can be found elsewhere (57, 66).

### Ferroptosis in Autoimmune Diseases

#### Ferroptosis in SLE

Systemic lupus erythematosus is a unique autoimmune disease triggered by the overproduction of a variety of autoantibodies directed against cellular components from the nucleus, cytoplasm and membrane, and the resulting formation and deposition of immune complexes in different tissues and organs can cause organ damage. The induction and exaggeration of cell death and impairment of the removal or uptake of dead cells by macrophages increases the opportunity to expose intracellular contents to the immune system and induce immune responses (67, 68). In the hemopoietic system, a decrease in immune cells, such as neutrophils, lymphocytes, red blood cells and platelets, is commonly observed in patients with SLE, although the mechanisms are not exactly clear. Studies have shown that peripheral blood neutrophils collected from pediatric SLE patients tend to die spontaneously and more quickly than healthy neutrophils in cell culture (69). A significant event accompanying neutrophil death is the induction of neutrophil extracellular trap (NET) release, which activates Toll-like receptors and induces the generation of ROS. NET is also a potent stimulant that induces the release of type I interferon (IFN) from plasmacytoid dendritic cells, which causes intense inflammatory reactions (69).

The mechanisms of cell death in neutrophils were recently demonstrated to be through ferroptosis manifesting with morphological changes, including vacuole formation, and the disappearance of cristae and increased membrane density in mitochondria (70). Reduced expression of Gpx4 in neutrophils can be readily detected in patients with SLE compared to healthy controls (**Table 2**). While treatment with serum immunoglobulin IgG from SLE patients or IFN-α causes a reduction in Gpx4 and neutrophil death, blockade of IFN-α resumes the survival of neutrophils (**Figure 1**). Treatment with ferroptosis inhibitors such as liproxstatin-1, a potent spiroquinoxalinamine derivative, and deferoxamine, an intracellular iron chelator, could attenuate neutrophil death induced by SLE patient serum. By analyzing the regulation of Gpx4 expression, the authors observed a conserved binding site in the Gpx4 promoter for cAMP-responsive element modulator α (CREMα), a tissue-specific transcriptional repressor. Further studies revealed increased nuclear accumulation and increased binding of CREMα to the Gpx4 promoter in SLE neutrophils, a phenomenon reproducibly detected by treating neutrophils from healthy individuals with SLE serum or IFN-α. The results are supportive for the observations of activated Ca^2+^/calmodulin–dependent kinase IV (CaMKIV)/CREMα axis in T lymphocytes of SLE patients (71). Insufficient CREMα brought about by genetic knockdown recovers the expression levels of Gpx4 (70).

**Table 2:** Summary of study findings of ferroptosis in autoimmune diseases.

	SLE (70)*	RA (81, 83)	IBD (90-100)
Source of the findings	Mice with myeloid-specific Gpx4 haploid deficiency and SLE patients	CIA mice model and joint synovium from RA patients	IECs in colonic mucosal samples from IBD patients and mice with DSS-induced colitis or preserving one Gpx4 allele
Methods of studies	Analyzing Gpx4 expression and neutrophil death and SLE parameters such as proteinuria and anti-ds DNA antibodies levels	Administering imidazole ketone erastin or RSL3 and examining synovial fibroblasts subsets and macrophages	Mice fed with PUFA-enriched diet like AA, or treated with DSS and examination on lipid peroxidation, IL-6 and CXCL1 production
Observations	Gpx4 haploid deficiency mice developed features characteristic of lupus, such as the production of anti-ds DNA antibodies, skin lesions and proteinuria. Neutrophils underwent ferroptosis in SLE patients.	Decrease of synovitis severity and prevention of arthritis and joint damage by induction of ferroptosis. TNF-α was protective against ferroptosis.	Ferroptosis of IECs from IBD patients and PUFA-fed Gpx4+/−IEC mice. Dietary AA induced production of IL-6 and CXCL1. Treatment with ferrostatin-1 attenuated DSS-induced colitis.
Mechanisms	IFN-α or SLE serum suppressed Gpx4 expression by increasing binding of CREMα to the Gpx4 promoter.	Macrophages released TNF-α to increase GSH biosynthesis and mediated protection of FAPα-positive synovial fibroblasts from undergoing ferroptosis.	Dietary AA administration reduced expression and enzymatic activity of Gpx4 and caused lipid peroxidation and ferroptosis in IECs.

*, references.

CIA, collagen-induced arthritis; anti-ds DNA, anti-double stranded DNA; IFN-α, interferon-alpha; DSS, dextran sodium sulfate; GSH, glutathione; RSL3, RAS synthetic lethal 3; CREMα, cAMP-responsive element modulator alpha; TNF-α, tumor necrosis factor-alpha; IEC, intestinal epithelial cells; IL-6, interleukin-6; CXCL1, chemokine (C-X-C motif) ligand 1; PUFA, polyunsaturated fatty acids; AA, arachidonic acid.

Strikingly, mice bearing myeloid-specific Gpx4 haploid deficiency developed several features characteristic of lupus, such as the production of anti-double stranded DNA (anti-ds DNA) antibodies, skin lesions and proteinuria and disease severity could be significantly reduced by the treatment with a specific ferroptosis inhibitor (70). Moreover, neutrophils from these genetically modified mice showed impaired viability and increased levels of lipid peroxidation (70). Somewhat unexpectedly, along with neutrophil ferroptosis in patients with SLE, there were no evident NETosis features in the neutrophils observed in this study, as suggested by Kenny et al. (70, 72). Although known as probably one of the most critical cytokines that mediates the pathogenic effects in SLE patients, how IFN-α mediates pathogenic processes remains largely unknown. The study by Li et al. demonstrated the critical roles of IFN-α in downregulating Gpx4 expression and inducing neutrophil ferroptosis in patients with SLE, highlighting the pathogenic roles and mechanisms of IFN-α in SLE (70). The roles of ferroptosis in SLE were also recently reviewed in a sophisticated manner (73). It is interesting to know whether blockade of neutrophil ferroptosis can show any therapeutic benefits in patients with SLE.

Knowing that ferroptosis is tightly correlated with iron dysregulation, studies have revealed a strong association between iron status and iron availability and SLE. To maintain iron homeostasis, ferroportin, the only known exporter controlling the release of iron, is responsible for the export of ferrous iron from the cytoplasm in macrophages (74). The regulation of ferroportin expression is complex and varies among different cell types, whereas the levels of membrane-associated ferroportin are regulated by its ligand, the liver-derived peptide hepcidin, which is also regulated by iron (74, 75). The binding of hepcidin to ferroportin results in endocytosis and ferroportin proteolysis in lysosomes; therefore, a low hepcidin concentration is an indicator of a high level of ferroportin in tissue cells. In contrast, an increase in hepcidin leads to the reduced release of iron from stores and causes insufficient iron availability. The proinflammatory cytokine IL-6, which is elevated in patients with SLE, can regulate hepcidin expression and induce iron retention by downregulating ferroportin in macrophages (76). In addition, the deposition of immune complexes in the glomerulus of the kidney stimulates inflammatory reactions and increases hepcidin expression (77). Notably, there is reduced iron availability in patients with SLE compared to healthy controls due to a decrease in the level of the iron transporter transferrin (78). Studies by Vanarsa et al. have suggested that serum transferrin concentrations are inversely correlated with disease activity in patients with SLE (79). Altogether, the effects and mechanisms disclosed by these studies highlight and support the notion that the dysregulation of iron metabolism or iron availability by, for example, IFN-α, can result in ferroptosis and lead to characteristic features such as neutropenia, proteinuria, and the production of anti-ds DNA antibodies in patients with SLE (70).

#### Ferroptosis of Synovial Fibroblasts and Rheumatoid Arthritis

Oxidative stress increases the production of ROS to activate immune cells, induce synovial proliferation and synovitis, and lead to joint destruction in patients with RA (80). An assumption has indicated that oxidative stress-mediated ROS generation may be protective for synovial cells to proliferate in inflamed joints. Examination of the hallmarks of lipid peroxidation in synovial tissues revealed increased levels of the oxidative DNA damage biomarker 8-OHdG and the lipid peroxide 4-hydroxynonenal, an α,β-unsaturated hydroxyalkenal, in patients with RA compared to those in patients with osteoarthritis (OA) (81). Similarly, the increased expression levels of 8-OHdG and 4-hydroxynonenal were observed in the inflamed joints of mice with collagen-induced arthritis (CIA). Moreover, increased iron levels in the synovial fluid of patients with RA correlated well with disease activity (81).

Consistent with the notion that suppression of synovial fibroblast ferroptosis is one of the mechanisms involved in maintaining the inflammation status in joints, administration of the lipid peroxidation and ferroptosis inducer imidazole ketone erastin (IKE) resulted in a decrease in synovitis severity and prevented the development of arthritis and joint damage in CIA mice (81). Experiments showed that IKE treatment selectively reduced the population of fibroblast activation protein-α (FAPα)-positive synovial fibroblasts that were undetectable under noninflammatory conditions but significantly increased in the inflamed synovium of CIA mice (81). Interestingly, treatment with the Gpx4 inhibitor RSL3 specifically increased cell death in fibroblast activation protein-α (FAP)α+ fibroblasts but not in macrophages, endothelial cells, T cells or B cells (81). The increased sensitivity to RSL-induced cell death in synovial fibroblasts prepared from inflamed synovial fluid compared to fibrocytes obtained from peripheral blood suggests that certain environmental factors inside inflamed joints are responsible for preventing synovial fibroblasts from undergoing ferroptosis and producing lipid ROS. Furthermore, a higher number of surviving FAPα+ fibroblasts in synovial areas were close to macrophages, suggesting that macrophages may protect FAPα+ fibroblasts from lipid peroxidation and ferroptosis induced by IKE treatment in CIA mice. By applying single-cell RNA sequencing, researchers identified different subsets of FAPα+ fibroblasts in the synoviums of RA patients and CIA mice. The ferroptosis-sensitive subset expressed higher levels of genes related to extracellular matrix formation and remodeling, and the ferroptosis-resistant subset expressed higher levels of genes associated with cellular proliferation (81).

**Figure 2:**
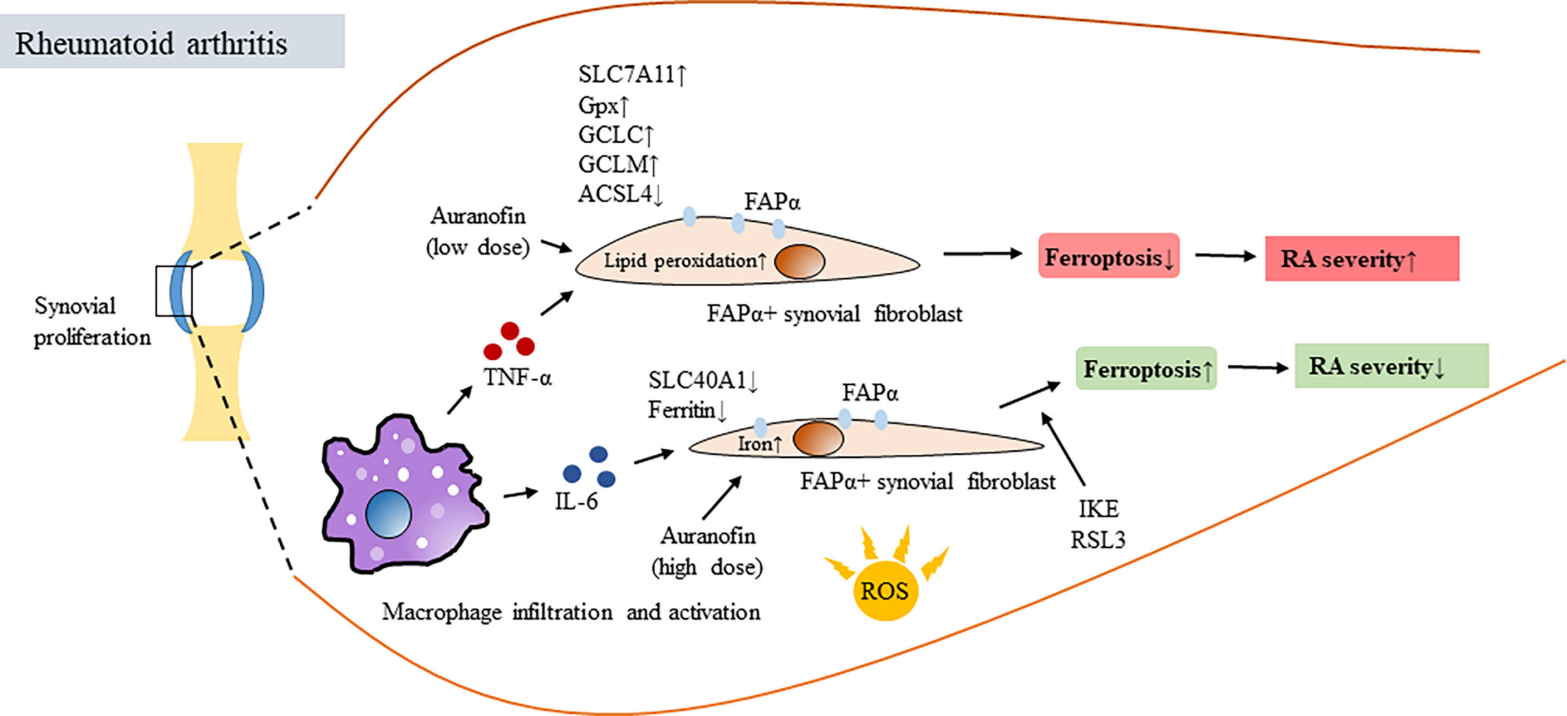
Ferroptosis of synovial fibroblasts in RA and its association with disease activity. Oxidative stress resulted in increased production of ROS, induced proliferation of synovial fibroblasts and caused synovitis and joint destruction in patients with RA. In synovial space, infiltrating macrophages produced proinflammatory cytokines such as TNF-α and IL-6, both are critical in mediating RA pathogenesis. However, TNF-α and IL-6 have different roles in regulating ferroptosis. TNF-α provided survival signal for fibroblast activation protein-α (FAP)α+ fibroblasts in synovial areas close to infiltrating macrophages, exaggerated synovial inflammation and increased RA disease activity. This was done by activating or inducing several regulators associated with GSH biosynthesis, including SLC7A11, GSH, NF-κB, GCLC, GCLM and reducing ACSL4 in ferroptosis-resistant FAPα+ fibroblasts. In contrast, IL-6 increased intracellular iron levels, decreased the expression of SLC40A1 and ferritin and resulted in inducing ferroptosis of synovial fibroblasts. Auranofin differential regulated ferroptosis depending on the dosages of administration. IKE and Gpx4 inhibitor RSL3 treatment induced ferroptosis, downregulated numbers of FAPα+ fibroblasts and reduced severity of synovitis. TNF-α, tuor necrosis factor alpha; IL-6, interleukin-6; FAP, fibroblast activation protein; Gpx4, glutathione peroxidase 4; SLC7A11, solute carrier family 7 member 11; GSH, glutathione; GCLC, a glutamate-cysteine ligase regulatory subunit; GCLM, a glutamate-cysteine ligase catalytic subunit; SLC40A1, solute carrier family 40 member 1; IKE, imidazole ketone erastin; RSL3, RAS synthetic lethal 3.

The protective effect provided by macrophages to ferroptosis-resistant FAPα+ fibroblasts in the synovium is likely due to the enriched production of TNF-α, a major proinflammatory cytokine that is mainly produced by macrophages and is a major contributor to the pathogenesis of RA (81, 82) (**Figure 2**). Genetic analysis of synovial fibroblasts from RA patients revealed that long-term treatment with TNF resulted in the increased expression of cell proliferation genes and the decreased expression of extracellular matrix-related genes (81). The dissection of cells with different lineages from RA hyperplastic synovium to examine the interplay among fibroblasts, macrophages, and TNF was then carried out. The results confirmed the existence of fibroblast subsets with distinct ferroptosis sensitivities that is likely due to reception of signaling of TNF released from activated macrophages (81). TNF-mediated protection against ferroptosis was also observed in human synovial fibroblasts treated with the ferroptosis inducers IKE and RSL3; however, the TNF-conferred protection from undergoing ferroptosis in fibroblasts was only observed at low doses and not at high doses (81). Treatment with IKE resulted in the depletion of GSH while TNF administration reversed this effect and increased the GSH levels in synovial fibroblasts. Treatment with TNF did not affect iron availability, whereas it did enhance the expression of several regulators associated with GSH biosynthesis, including solute carrier family 7 member 11 (SLC7A11), a functional subunit of system Xc- and a molecule responsible for the generation of intracellular GSH, GCLC (a glutamate-cysteine ligase regulatory subunit) and GCLM (a glutamate-cysteine ligase catalytic subunit) (81). It would also be interesting to know whether the therapeutic benefits of anti-TNF biologics may be attributed in part to blocking the TNF-mediated protection of synovial fibroblasts in the inflamed joints of RA patients.

The ferroptosis-protective effects of TNF on synovial fibroblasts appear to be specific because the effects were not observed for other proinflammatory cytokines, such as IL-6, which is also highly induced in inflamed joints in RA. In contrast, under similar conditions, IL-6 treatment increased intracellular iron levels, decreased the expression of solute carrier family 40 member 1 (SLC40A1) and ferritin and yet without changing the GSH levels, and resulted in the sensitization of fibroblasts to ferroptosis (81). This study further demonstrated that the combination of a low dose of IKE to deprive cystine and a low dose of the TNF blocker etanercept provided a synergistic effect to reduce p-NF-κB, GCLM, and GCLC expression by inducing fibroblast ferroptosis in CIA mice (81).

Furthermore, a reduction in ferroptosis accompanied by a decrease in acyl coenzyme A synthetase long chain 4 (ACSL4) and increases in Gpx4, ferritin heavy chain 1 and SLC7A11 was observed in the synoviums and synovial fibroblasts from RA patients compared to those from healthy controls (81). RNA-Seq analysis revealed that glycine could regulate the ferroptosis pathway by upregulating the S-adenosylmethionine (SAM) concentration and downregulating Gpx4 expression by inducing SAM-mediated Gpx4 promoter methylation in RA synovial fibroblasts (83). Altogether, these studies (81, 83) suggest that inducing ferroptosis in proliferating synovial fibroblasts in the inflamed joints of RA patients may be therapeutically beneficial. Regarding this, the older anti-RA mediation auranofin can also serve as an example. Auranofin contains gold and was used to treat patients with RA (84); however, because of its high toxicity and the great advancements in modern therapeutics, this medication is no longer used to treat patients with RA, although it remains a potential treatment for other diseases (85). Given that high-dose auranofin treatment (25 mg/kg) caused lipid peroxidation and ferroptosis by inhibiting thioredoxin reductase (TXNRD) activity, treatment with a low dose (5 mg/kg) potently induced the expression of hepcidin by activating the bone morphogenetic protein (BMP)/Smad and IL-6/Janus kinase (Jak)2/signal transducer and activator of transcription (Stat)3 pathways, leading to reduced serum iron, transferrin saturation and inhibition of ferroptosis in mice (86). Aiming to regulate cellular ferroptosis while maintaining tolerable drug toxicity, choosing a therapeutic dose of auranofin appears to be critical. Given the critical process of ROS production in RA pathogenesis, ferroptosis-targeted therapy has been proposed (87, 88).

#### Ferroptosis and Inflammatory Bowel Diseases

**Figure 3:**
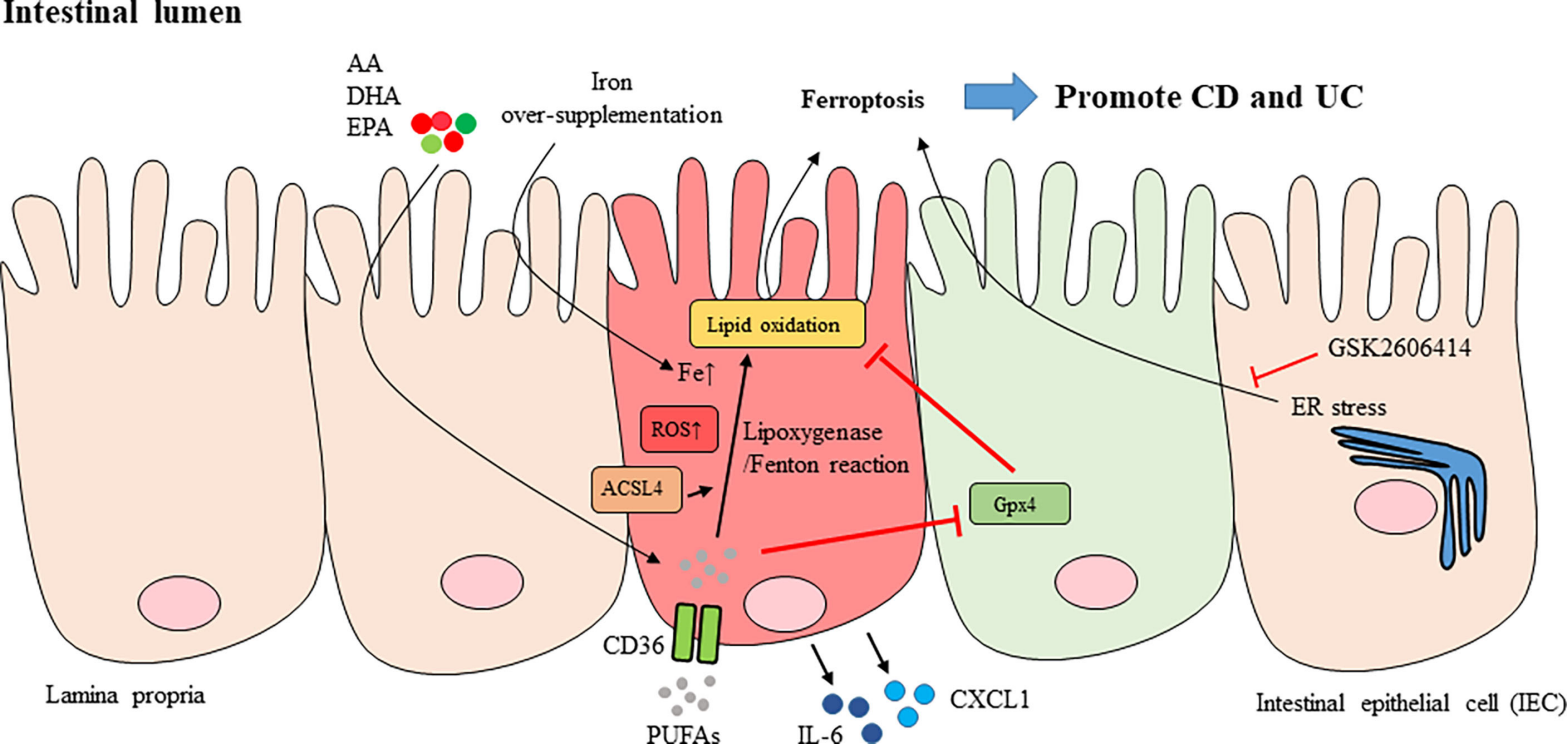
Ferroptosis of intestinal epithelial cells in IBD. Over-supplementation of iron caused its deposition in the intestine and led to excessive production of ROS and intestinal inflammation (red IEC). AA, EPA and DHA listed here are part of PUFAs. PUFAs whose uptake is mediated by CD36 are the major substrates for lipid peroxidation. The oxidation of PUFAs can be regulated by either lipoxygenase-mediated reaction, or Fenton-type reaction, in the plasma membrane. ACSL4 that esterifies AA into phospholipids facilitated peroxidation. Oxidation of PUFAs induced pro-inflammatory cytokines or mediators like IL-6, CXCL1 production in IEC as well as lipid peroxidation and ferroptosis of IECs. The expression and enzymatic activity of Gpx4 prevents lipid peroxidation in IEC (green IEC). The ER stress was also shown to be mediating IEC ferroptosis and suppression of ER stress with GSK2606414 inhibited ferroptosis of IECs. CD, Crohn’s disease; UC, ulcerative colitis; IEC, intestinal epithelial cell; PUFAs, polyunsaturated fatty acids; AA: arachidonic acid; EPA, eicosapentaenoic acid; DHA, docosahexaenoic acid; GSH, glutathione; Gpx4, glutathione peroxidase 4; ACSL4, acyl coenzyme A synthetase long chain 4; ER, endoplasmic reticulum; IL-6, interleukin-6; CXCL1, chemokine (C-X-C motif) ligand 1.

As the intestinal epithelium is a critical organ for nutrient absorption, cell death here must be carefully regulated, and any interference with cell regeneration or cell death processes may cause pathologies. The inflammatory bowel diseases Crohn’s disease (CD) and ulcerative colitis (UC) manifest with more cell death in the intestine and colon due to chronic inflammation. Several cell death subtypes have been shown to participate in causing chronic intestinal inflammation (89). Over-supplementation of iron increases its deposition in the intestine, resulting in excessive production of ROS and inducing intestinal inflammation in dextran sodium sulfate (DSS)-induced colitis in mice, an animal model of IBD (90, 91) (**Figure 3**). Indeed, iron supplementation may have either beneficial or detrimental effects by influencing the gut microbiota (92). Additionally, supported by studies showing the crucial roles of ROS and iron in the progression of UC (93), colonic mucosal specimens from UC patients expressed genetic patterns compatible with those in ferroptosis (94). Similar features were also observed in experimental colitis in mice treated with DSS (94). In addition, treatment with the ferroptosis inhibitor ferrostatin-1 attenuated experimental colitis induced by DSS administration (94).

Polyunsaturated fatty acids (PUFAs), especially arachidonic acid (AA) and linoleic acid, preserve highly oxidizable methylene groups that bridge two double bonds and are the two major substrates for lipid peroxidation (66, 95). Compared to the less reactive monounsaturated and saturated fatty acids, PUFAs are ideal targets for attack by ROS, and accumulated cytosolic and lipid ROS serve as potent triggers to oxidize PUFAs to generate lipid peroxides, thus inducing ferroptosis (10, 96). The oxidation of PUFAs can be regulated by either an enzymatic reaction, such as a lipoxygenase-mediated reaction, or a nonenzymatic reaction, such as a Fenton-type reaction, in the plasma membrane (57, 97). Exposure to a PUFA-enriched Western diet induced lipid peroxidation and intestinal inflammation in mice with the specific deletion of one Gpx4 allele, which resulted in an ~50% reduction in Gpx4 mRNA and protein levels in the intestinal epithelial cells (IECs) of both the large and small intestines compared to wild-type mice (97). In addition, the oral uptake of AA resulted in neutrophil infiltration in the small intestines of iron-primed mice with IECs deficient in one Gpx4 allele (97). Certain features, including neutrophil infiltration into mucosal and submucosal lesions, epithelial injury, crypt hyperplasia, and granuloma-like accumulation of inflammatory cells reminiscent of certain small intestinal CD characteristics, were observed in PUFA WD-fed Gpx4+/−IEC mice (97). In addition, epithelial lipid peroxidation and the increased expression of chemokine (C-X-C motif) ligand 1 (CXCL1) were noted in the lesions. However, there was no evident IEC death in Gpx4+/−IEC mice fed a PUFA-enriched Western diet, suggesting that cell death was not absolutely required for PUFA-induced intestinal inflammation in Gpx4+/−IEC mice (97). Ferric iron promoted lipid peroxidation and AA-induced IL-6 and CXCL1 production in siGpx4 IECs, whereas treatment with the iron chelator deferoxamine or the lipid peroxidation scavenger ferrostatin-1 and α-tocopherol or inhibition of lipoxygenases (LOX)15 reduced IL-6 and CXCL1 production in AA-stimulated siGpx4 IECs (97). These data suggested that iron availability is critical to control PUFA-induced lipid peroxidation and cytokine production in IECs with reduced Gpx4 levels and enzymatic activity.

In patients with active UC, AA accumulates in the colonic mucosa (98), which correlates with evidence showing that increased uptake of dietary AA increases the risk of UC (99). Since ACSL4, an enzyme that ligates long PUFAs to coenzyme A, esterifies AA into phospholipids to facilitate peroxidation (7, 100), subsequent experiments demonstrated that AA-induced IL-6 and CXCL1 production was blocked in Acsl4-deleted siGpx4 IECs (97). In parallel, AA stimulation induced lipid peroxidation, which was not inhibited by Acsl4 deletion in siGpx4 IECs. These results indicate that ACSL4 and Gpx4 may differentially regulate AA-induced cytokine responses and lipid peroxidation, respectively (97). In addition, reduced expression and decreased enzymatic activity of Gpx4 and lipid peroxidation in IECs derived from the lesioned small intestinal mucosa of patients with CD were observed (97). Production of the proinflammatory cytokine IL-6 and the chemokine CXCL1 was induced by treatment with PUFAs, specifically AA, in IECs showing reduced Gpx4 activity. Arachidonic acid treatment also induced the expression of IL-6 and CXCL1 in IECs treated with siGpx4 but not those treated with the control reagent (97). Similar effects were observed in IECs treated with different ω-3 and ω-6 PUFAs, including stearidonic acid, docosahexaenoic acid, eicosapentaenoic acid and docosapentaenoic acid, all of which also induce lipid peroxidation and CXCL1 and IL-6 production (97). The conclusions from these studies are consistent with a meta-analysis incorporating CD and UC genome-wide association scans that revealed a genetic association between Gpx4 and CD (101).

### Perspective and Potential of Targeting Ferroptosis for Autoimmune Disease Therapeutics

As indicated, how ferroptosis is initiated and executed remains largely unclear. Several inducers/sensitizers such as erastin or its analogues, drugs like sulfasalazine, sorafenib and statins, and high concentration of extracellular glutamate could serve to trigger ferroptosis (57). However, there is no specific receptors involved in response to these potential ferroptosis inducers/sensitizers (57). Our current understanding about ferroptosis is that ferroptosis is induced by the dysregulation of molecules critically controlling redox systems and preventing lipid peroxidation (66). Different tissue cells may show differential sensitivity to ferroptosis and cancer cells seem to be very sensitive to lipid peroxidation (21). As an example, in SLE, ferroptosis happened only in neutrophils but not in monocytes or lymphocytes, a phenomenon likely due to the relatively lower levels of Gpx4 in neutrophils compared to that in monocytes and lymphocytes in healthy people (70). The downstream signaling molecules and signaling pathways involved in mediating ferroptosis is also not clear. Nevertheless, several critical molecules that are of potential to regulate ferroptosis processes have been identified. By using a synthetic lethal CRISPR-Cas9 screen, the researchers identified ferroptosis suppressor protein 1 (FSP1), also known as apoptosis-inducing factor mitochondria associated 2 (AIFM2), as a potent ferroptosis-resistance molecule that effectively prevented ferroptosis in a GSH-independent manner. To be effective, FSP1 must be recruited to the plasma membrane *via* its myristylation binding motifs and mediate oxidoreductase activity to reduce coenzyme Q_10_ (CoQ), a lipophilic radical-trapping antioxidant halting the propagation of lipid peroxides (102, 103). Applying RNA-seq approaches, Brown et al. identified a pentaspanin protein prominin 2, a lipid dynamic regulator, that could promote the formation of ferritin-containing multivesicular bodies and exosomes and mediate transportation of iron out of the cell to inhibit ferroptosis (104). We refer the readers to an excellent review by Stockwell et al. (57) regarding the inducers, inhibitors and genes involved in ferroptosis as well as its association with metabolism. 

In terms of therapeutics, targeting ferroptosis for the treatment of autoimmune diseases appears to be complicated because ferroptotic processes occurring in different diseases and different tissues may have different impacts. As discussed above, while the induction of ferroptosis in the synovium of patients with RA may be therapeutically beneficial, cell survival preservation and ferroptosis inhibition or prevention may be favorable for treating diseases such as IBD and SLE. In addition, given that the factors that induce ferroptosis in these different autoimmune diseases remain largely unclear, cytokines such as TNF-α and IFN-α regulate ferroptosis in different manners and contribute in various ways to disease pathogenesis (70, 81). While IL-6 may be detrimental by causing ferroptosis in IECs (97), the ferroptosis-inducing effects of IL-6 may be beneficial for controlling joint inflammation in RA patients (81). Furthermore, as the example in SLE patients shows, under exposure to the same extracellular conditions, IFN-α-induced ferroptosis occurred only in neutrophils but not in lymphocytes or monocytes (70). All these clues make targeting ferroptosis for therapeutic purposes even more complicated. It is evident that scientific societies need more studies to examine ferroptosis events and the underlying mechanisms that occur in autoimmune diseases. Whether a beneficial or a detrimental outcome is achieved by manipulating cellular ferroptosis will be dependent on the results from animal studies and clinical trials.

Practically, there are many different approaches to block ferroptosis: pharmacologically using thiazolidinediones to inhibit ACSL4 activity, restoring the level of the key ferroptosis regulator Gpx4, administering ferroptosis-specific inhibitors such as ferrostatins and liproxstatins, preventing lipid peroxidation with supplementation of fatty acids, iron chelators and antioxidants, or modulating metabolic pathways (53). As an example, one antirheumatic drug, sulfasalazine, is a ferroptosis inducer. Sulfasalazine suppresses GSH biosynthesis and inhibits system Xc^-^ to induce ferroptosis in tumors (4, 105). However, it remains unclear whether the pro-ferroptotic effects of sulfasalazine may account for the observed effects in autoimmune disorders such as RA and spondyloarthritis. Given that ER stress was shown to mediate IEC ferroptosis in colonic mucosal specimens from UC patients, inhibiting ER stress with GSK2606414 could prevent IECs from undergoing ferroptosis and reduce the severity of experimental colitis in mice (94). Furthermore, treatment with OTSSP167, a MELK-selective inhibitor, significantly inhibited ferroptosis and reduced DSS-induced colitis in mice by suppressing the protein kinase B (AKT)/IKK/p65 and extracellular signal-regulated kinase (ERK)/IKK/p65 signaling cascades (106).

Knowing that apoptosis and other cell death subtypes are also observed in IECs from patients with UC and CD (107–111), how these different cell death pathways interact with one another and the consequences of these interactions in autoimmune diseases are interesting topics to investigate. In addition, how cellular mechanisms regulate these different cell death subtypes to either maintain homeostasis or counteract threats from extracellular stress or pathogens is largely unknown and deserves further investigation. Altogether, studies targeting ferroptosis events, effects, mechanisms, and therapeutics will be hot topics to pursue in the field of autoimmune diseases in the future.

## Author Contributions

Writing—original draft preparation, BL. and J-HL. Writing—review and editing, BL, C-YW, C-HW, S-FL and J-HL. Supervision, J-HL. Funding acquisition, J-HL. All authors contributed to the article and approved the submitted version.

## Funding

This work was supported by grants from the Ministry of Science and Technology (MOST 110-2314-B-182A-127-MY3) and Chang Gung Memorial Hospital (CMRPG1M0021), Taiwan, R.O.C.

## Conflict of Interest

The authors declare that the research was conducted in the absence of any commercial or financial relationships that could be construed as a potential conflict of interest.

## Publisher’s Note

All claims expressed in this article are solely those of the authors and do not necessarily represent those of their affiliated organizations, or those of the publisher, the editors and the reviewers. Any product that may be evaluated in this article, or claim that may be made by its manufacturer, is not guaranteed or endorsed by the publisher.

## References

[1] Dolma S Lessnick SL Hahn WC Stockwell BR. Identification of Genotype-Selective Antitumor Agents Using Synthetic Lethal Chemical Screening in Engineered Human Tumor Cells. Cancer Cell (2003) 3(3):285–96. doi: 10.1016/s1535-6108(03)00050-3 12676586

[2] Yang WS Stockwell BR. Synthetic Lethal Screening Identifies Compounds Activating Iron-Dependent, Nonapoptotic Cell Death in Oncogenic-RAS-Harboring Cancer Cells. Chem Biol (2008) 15(3):234–45. doi: 10.1016/j.chembiol.2008.02.010 PMC2683762 18355723

[3] Yagoda N von Rechenberg M Zaganjor E Bauer AJ Yang WS Fridman DJ. RAS-RAF-MEK-Dependent Oxidative Cell Death Involving Voltage-Dependent Anion Channels. Nature (2007) 447(7146):864–8. doi: 10.1038/nature05859 PMC3047570 17568748

[4] Dixon SJ Lemberg KM Lamprecht MR Skouta R Zaitsev EM Gleason CE. Ferroptosis: An Iron-Dependent Form of Nonapoptotic Cell Death. Cell (2012) 149(5):1060–72. doi: 10.1016/j.cell.2012.03.042 PMC3367386 22632970

[5] Friedmann Angeli JP Schneider M Proneth B Tyurina YY Tyurin VA Hammond VJ. Inactivation of the Ferroptosis Regulator Gpx4 Triggers Acute Renal Failure in Mice. Nat Cell Biol (2014) 16(12):1180–91. doi: 10.1038/ncb3064 PMC4894846 25402683

[6] Kim SE Zhang L Ma K Riegman M Chen F Ingold I. Ultrasmall Nanoparticles Induce Ferroptosis in Nutrient-Deprived Cancer Cells and Suppress Tumour Growth. Nat Nanotechnol (2016) 11(11):977–85. doi: 10.1038/nnano.2016.164 PMC5108575 27668796

[7] Doll S Proneth B Tyurina YY Panzilius E Kobayashi S Ingold I. ACSL4 Dictates Ferroptosis Sensitivity by Shaping Cellular Lipid Composition. Nat Chem Biol (2017) 13(1):91–8. doi: 10.1038/nchembio.2239 PMC5610546 27842070

[8] Chen X Kang R Kroemer G Tang D. Organelle-Specific Regulation of Ferroptosis. Cell Death Differ (2021) 28(10):2843–56. doi: 10.1038/s41418-021-00859-z PMC8481335 34465893

[9] Jiang X Stockwell BR Conrad M. Ferroptosis: Mechanisms, Biology and Role in Disease. Nat Rev Mol Cell Biol (2021) 22(4):266–82. doi: 10.1038/s41580-020-00324-8 PMC8142022 33495651

[10] Xu C Liu Z Xiao J. Ferroptosis: A Double-Edged Sword in Gastrointestinal Disease. Int J Mol Sci (2021) 22(22):ijms222212403. doi: 10.3390/ijms222212403 PMC8620748 34830285

[11] Rodriguez R Schreiber SL Conrad M. Persister Cancer Cells: Iron Addiction and Vulnerability to Ferroptosis. Mol Cell (2022) 82(4):728–40. doi: 10.1016/j.molcel.2021.12.001 PMC9152905 34965379

[12] Nagata S. Apoptosis and Clearance of Apoptotic Cells. Annu Rev Immunol (2018) 36:489–517. doi: 10.1146/annurev-immunol-042617-053010 29400998

[13] Guo C Fu R Zhou M Wang S Huang Y Hu H. Pathogenesis of Lupus Nephritis: RIP3 Dependent Necroptosis and NLRP3 Inflammasome Activation. J Autoimmun (2019) 103:102286. doi: 10.1016/j.jaut.2019.05.014 31133359 PMC6708470

[14] Fan H Liu F Dong G Ren D Xu Y Dou J. Activation-Induced Necroptosis Contributes to B-Cell Lymphopenia in Active Systemic Lupus Erythematosus. Cell Death Dis (2014) 5:e1416. doi: 10.1038/cddis.2014.375 25210799 PMC4225223

[15] Zhao J Jiang P Guo S Schrodi SJ He D. Apoptosis, Autophagy, NETosis, Necroptosis, and Pyroptosis Mediated Programmed Cell Death as Targets for Innovative Therapy in Rheumatoid Arthritis. Front Immunol (2021) 12:809806. doi: 10.3389/fimmu.2021.809806 35003139 PMC8739882

[16] Wang R Li H Wu J Cai ZY Li B Ni H. Gut Stem Cell Necroptosis by Genome Instability Triggers Bowel Inflammation. Nature (2020) 580(7803):386–90. doi: 10.1038/s41586-020-2127-x 32296174

[17] Chen X Liu G Yuan Y Wu G Wang S Yuan L. NEK7 Interacts With NLRP3 to Modulate the Pyroptosis in Inflammatory Bowel Disease *via* NF-kappaB Signaling. Cell Death Dis (2019) 10(12):906. doi: 10.1038/s41419-019-2157-1 31787755 PMC6885517

[18] Lichtenberger GS Flavell RA Alexopoulou L. Innate Immunity and Apoptosis in IBD. Inflamm Kr Bowel Dis (2004) 10 Suppl 1:S58–62. doi: 10.1097/00054725-200402001-00012 15168833

[19] Hu CL Nydes M Shanley KL Morales Pantoja IE Howard TA Bizzozero OA. Reduced Expression of the Ferroptosis Inhibitor Glutathione Peroxidase-4 in Multiple Sclerosis and Experimental Autoimmune Encephalomyelitis. J Neurochem (2019) 148(3):426–39. doi: 10.1111/jnc.14604 PMC6347488 30289974

[20] Duprez L Wirawan E Vanden Berghe T Vandenabeele P. Major Cell Death Pathways at a Glance. Microbes Infect (2009) 11(13):1050–62. doi: 10.1016/j.micinf.2009.08.013 19733681

[21] Tonnus W Belavgeni A Beuschlein F Eisenhofer G Fassnacht M Kroiss M. The Role of Regulated Necrosis in Endocrine Diseases. Nat Rev Endocrinol (2021) 17(8):497–510. doi: 10.1038/s41574-021-00499-w 34135504 PMC8207819

[22] Peter ME. Programmed Cell Death: Apoptosis Meets Necrosis. Nature (2011) 471(7338):310–2. doi: 10.1038/471310a 21412328

[23] Roberts JZ Crawford N Longley DB. The Role of Ubiquitination in Apoptosis and Necroptosis. Cell Death Differ (2022) 29(2):272–84. doi: 10.1038/s41418-021-00922-9 PMC8817035 34912054

[24] Chipuk JE Bouchier-Hayes L Green DR. Mitochondrial Outer Membrane Permeabilization During Apoptosis: The Innocent Bystander Scenario. Cell Death Differ (2006) 13(8):1396–402. doi: 10.1038/sj.cdd.4401963 16710362

[25] Susin SA Daugas E Ravagnan L Samejima K Zamzami N Loeffler M. Two Distinct Pathways Leading to Nuclear Apoptosis. J Exp Med (2000) 192(4):571–80. doi: 10.1084/jem.192.4.571 PMC2193229 10952727

[26] Kerr JF Wyllie AH Currie AR. Apoptosis: A Basic Biological Phenomenon With Wide-Ranging Implications in Tissue Kinetics. Br J Cancer (1972) 26(4):239–57. doi: 10.1038/bjc.1972.33 PMC2008650 4561027

[27] Miyake S Murai S Kakuta S Uchiyama Y Nakano H. Identification of the Hallmarks of Necroptosis and Ferroptosis by Transmission Electron Microscopy. Biochem Biophys Res Commun (2020) 527(3):839–44. doi: 10.1016/j.bbrc.2020.04.127 32430176

[28] Vanden Berghe T Vanlangenakker N Parthoens E Deckers W Devos M Festjens N. Necroptosis, Necrosis and Secondary Necrosis Converge on Similar Cellular Disintegration Features. Cell Death Differ (2010) 17(6):922–30. doi: 10.1038/cdd.2009.184 20010783

[29] Vanden Berghe T Linkermann A Jouan-Lanhouet S Walczak H Vandenabeele P. Regulated Necrosis: The Expanding Network of non-Apoptotic Cell Death Pathways. Nat Rev Mol Cell Biol (2014) 15(2):135–47. doi: 10.1038/nrm3737 24452471

[30] Wang H Sun L Su L Rizo J Liu L Wang LF. Mixed Lineage Kinase Domain-Like Protein MLKL Causes Necrotic Membrane Disruption Upon Phosphorylation by RIP3. Mol Cell (2014) 54(1):133–46. doi: 10.1016/j.molcel.2014.03.003 24703947

[31] Yoon S Bogdanov K Kovalenko A Wallach D. Necroptosis is Preceded by Nuclear Translocation of the Signaling Proteins That Induce it. Cell Death Differ (2016) 23(2):253–60. doi: 10.1038/cdd.2015.92 PMC4716306 26184911

[32] Lalaoui N Boyden SE Oda H Wood GM Stone DL Chau D. Mutations That Prevent Caspase Cleavage of RIPK1 Cause Autoinflammatory Disease. Nature (2020) 577(7788):103–8. doi: 10.1038/s41586-019-1828-5 PMC6930849 31827281

[33] Tao P Sun J Wu Z Wang S Wang J Li W. A Dominant Autoinflammatory Disease Caused by non-Cleavable Variants of RIPK1. Nature (2020) 577(7788):109–14. doi: 10.1038/s41586-019-1830-y 31827280

[34] Jesenberger V Procyk KJ Yuan J Reipert S Baccarini M. Salmonella-Induced Caspase-2 Activation in Macrophages: A Novel Mechanism in Pathogen-Mediated Apoptosis. J Exp Med (2000) 192(7):1035–46. doi: 10.1084/jem.192.7.1035 PMC2193309 11015444

[35] Jorgensen I Zhang Y Krantz BA Miao EA. Pyroptosis Triggers Pore-Induced Intracellular Traps (PITs) That Capture Bacteria and Lead to Their Clearance by Efferocytosis. J Exp Med (2016) 213(10):2113–28. doi: 10.1084/jem.20151613 PMC5030797 27573815

[36] Galluzzi L Vitale I Aaronson SA Abrams JM Adam D Agostinis P. Molecular Mechanisms of Cell Death: Recommendations of the Nomenclature Committee on Cell Death 2018. Cell Death Differ (2018) 25(3):486–541. doi: 10.1038/s41418-017-0012-4 29362479 PMC5864239

[37] Yu J Nagasu H Murakami T Hoang H Broderick L Hoffman HM. Inflammasome Activation Leads to Caspase-1-Dependent Mitochondrial Damage and Block of Mitophagy. Proc Natl Acad Sci U S A (2014) 111(43):15514–9. doi: 10.1073/pnas.1414859111 PMC4217429 25313054

[38] Bergsbaken T Fink SL Cookson BT. Pyroptosis: Host Cell Death and Inflammation. Nat Rev Microbiol (2009) 7(2):99–109. doi: 10.1038/nrmicro2070 19148178 PMC2910423

[39] Jorgensen I Miao EA. Pyroptotic Cell Death Defends Against Intracellular Pathogens. Immunol Rev (2015) 265(1):130–42. doi: 10.1111/imr.12287 PMC4400865 25879289

[40] Evavold CL Hafner-Bratkovic I Devant P D’Andrea JM Ngwa EM Borsic E. Control of Gasdermin D Oligomerization and Pyroptosis by the Ragulator-Rag-Mtorc1 Pathway. Cell (2021) 184(17):4495–511.e19. doi: 10.1016/j.cell.2021.06.028 34289345 PMC8380731

[41] Baroja-Mazo A Martin-Sanchez F Gomez AI Martinez CM Amores-Iniesta J Compan V. The NLRP3 Inflammasome is Released as a Particulate Danger Signal That Amplifies the Inflammatory Response. Nat Immunol (2014) 15(8):738–48. doi: 10.1038/ni.2919 24952504

[42] Schwarzer R Laurien L Pasparakis M. New Insights Into the Regulation of Apoptosis, Necroptosis, and Pyroptosis by Receptor Interacting Protein Kinase 1 and Caspase-8. Curr Opin Cell Biol (2020) 63:186–93. doi: 10.1016/j.ceb.2020.02.004 32163825

[43] Das S Shukla N Singh SS Kushwaha S Shrivastava R. Mechanism of Interaction Between Autophagy and Apoptosis in Cancer. Apoptosis (2021) 26(9-10):512–33. doi: 10.1007/s10495-021-01687-9 34510317

[44] Gao W Zhang T Wu H. Emerging Pathological Engagement of Ferroptosis in Gut Diseases. Oxid Med Cell Longev (2021) 2021:4246255. doi: 10.1155/2021/4246255 34733403 PMC8560274

[45] Magtanong L Ko PJ Dixon SJ. Emerging Roles for Lipids in non-Apoptotic Cell Death. Cell Death Differ (2016) 23(7):1099–109. doi: 10.1038/cdd.2016.25 PMC5399169 26967968

[46] Chen X Comish PB Tang D Kang R. Characteristics and Biomarkers of Ferroptosis. Front Cell Dev Biol (2021) 9:637162. doi: 10.3389/fcell.2021.637162 33553189 PMC7859349

[47] Yan B Ai Y Sun Q Ma Y Cao Y Wang J. Membrane Damage During Ferroptosis Is Caused by Oxidation of Phospholipids Catalyzed by the Oxidoreductases POR and CYB5R1. Mol Cell (2021) 81(2):355–69.e10. doi: 10.1016/j.molcel.2020.11.024 33321093

[48] Lawen A Lane DJ. Mammalian Iron Homeostasis in Health and Disease: Uptake, Storage, Transport, and Molecular Mechanisms of Action. Antioxid Redox Signal (2013) 18(18):2473–507. doi: 10.1089/ars.2011.4271 23199217

[49] Muckenthaler MU Rivella S Hentze MW Galy B. A Red Carpet for Iron Metabolism. Cell (2017) 168(3):344–61. doi: 10.1016/j.cell.2016.12.034 PMC5706455 28129536

[50] Golonka R Yeoh BS Vijay-Kumar M. The Iron Tug-Of-War Between Bacterial Siderophores and Innate Immunity. J Innate Immun (2019) 11(3):249–62. doi: 10.1159/000494627 PMC6487204 30605903

[51] Zhang C. Essential Functions of Iron-Requiring Proteins in DNA Replication, Repair and Cell Cycle Control. Protein Cell (2014) 5(10):750–60. doi: 10.1007/s13238-014-0083-7 PMC4180463 25000876

[52] Gutteridge JM Halliwell B. Iron Toxicity and Oxygen Radicals. Baillieres Clin Haematol (1989) 2(2):195–256. doi: 10.1016/s0950-3536(89)80017-4 2660928

[53] Doll S Conrad M. Iron and Ferroptosis: A Still Ill-Defined Liaison. IUBMB Life (2017) 69(6):423–34. doi: 10.1002/iub.1616 28276141

[54] Pantopoulos K Porwal SK Tartakoff A Devireddy L. Mechanisms of Mammalian Iron Homeostasis. Biochemistry (2012) 51(29):5705–24. doi: 10.1021/bi300752r PMC3572738 22703180

[55] Yang WS SriRamaratnam R Welsch ME Shimada K Skouta R Viswanathan VS. Regulation of Ferroptotic Cancer Cell Death by GPX4. Cell (2014) 156(1-2):317–31. doi: 10.1016/j.cell.2013.12.010 PMC4076414 24439385

[56] Ursini F Maiorino M Gregolin C. The Selenoenzyme Phospholipid Hydroperoxide Glutathione Peroxidase. Biochim Biophys Acta (1985) 839(1):62–70. doi: 10.1016/0304-4165(85)90182-5 3978121

[57] Stockwell BR Friedmann Angeli JP Bayir H Bush AI Conrad M Dixon SJ. Ferroptosis: A Regulated Cell Death Nexus Linking Metabolism, Redox Biology, and Disease. Cell (2017) 171(2):273–85. doi: 10.1016/j.cell.2017.09.021 PMC5685180 28985560

[58] Seiler A Schneider M Forster H Roth S Wirth EK Culmsee C. Glutathione Peroxidase 4 Senses and Translates Oxidative Stress Into 12/15-Lipoxygenase Dependent- and AIF-Mediated Cell Death. Cell Metab (2008) 8(3):237–48. doi: 10.1016/j.cmet.2008.07.005 18762024

[59] Griffith OW. Biologic and Pharmacologic Regulation of Mammalian Glutathione Synthesis. Free Radic Biol Med (1999) 27(9-10):922–35. doi: 10.1016/s0891-5849(99)00176-8 10569625

[60] Dickinson DA Forman HJ. Cellular Glutathione and Thiols Metabolism. Biochem Pharmacol (2002) 64(5-6):1019–26. doi: 10.1016/s0006-2952(02)01172-3 12213601

[61] Bannai S. Exchange of Cystine and Glutamate Across Plasma Membrane of Human Fibroblasts. J Biol Chem (1986) 261(5):2256–63. doi: 10.1016/S0021-9258(17)35926-4 2868011

[62] Sasaki H Sato H Kuriyama-Matsumura K Sato K Maebara K Wang H. Electrophile Response Element-Mediated Induction of the Cystine/Glutamate Exchange Transporter Gene Expression. J Biol Chem (2002) 277(47):44765–71. doi: 10.1074/jbc.M208704200 12235164

[63] Itoh K Tong KI Yamamoto M. Molecular Mechanism Activating Nrf2-Keap1 Pathway in Regulation of Adaptive Response to Electrophiles. Free Radic Biol Med (2004) 36(10):1208–13. doi: 10.1016/j.freeradbiomed.2004.02.075 15110385

[64] Bannai S Sato H Ishii T Sugita Y. Induction of Cystine Transport Activity in Human Fibroblasts by Oxygen. J Biol Chem (1989) 264(31):18480–4. doi: 10.1016/S0021-9258(18)51491-5 2808385

[65] Davidson AJ Wood W. Igniting the Spread of Ferroptotic Cell Death. Nat Cell Biol (2020) 22(9):1027–9. doi: 10.1038/s41556-020-0570-4 32868901

[66] Conrad M Pratt DA. The Chemical Basis of Ferroptosis. Nat Chem Biol (2019) 15(12):1137–47. doi: 10.1038/s41589-019-0408-1 31740834

[67] Bijl M Reefman E Horst G Limburg PC Kallenberg CG. Reduced Uptake of Apoptotic Cells by Macrophages in Systemic Lupus Erythematosus: Correlates With Decreased Serum Levels of Complement. Ann Rheum Dis (2006) 65(1):57–63. doi: 10.1136/ard.2005.035733 15919679 PMC1797975

[68] Tas SW Quartier P Botto M Fossati-Jimack L. Macrophages From Patients With SLE and Rheumatoid Arthritis Have Defective Adhesion *In Vitro*, While Only SLE Macrophages Have Impaired Uptake of Apoptotic Cells. Ann Rheum Dis (2006) 65(2):216–21. doi: 10.1136/ard.2005.037143 PMC1798004 16014673

[69] Garcia-Romo GS Caielli S Vega B Connolly J Allantaz F Xu Z. Netting Neutrophils are Major Inducers of Type I IFN Production in Pediatric Systemic Lupus Erythematosus. Sci Transl Med (2011) 3(73):73ra20. doi: 10.1126/scitranslmed.3001201 PMC3143837 21389264

[70] Li P Jiang M Li K Li H Zhou Y Xiao X. Glutathione Peroxidase 4-Regulated Neutrophil Ferroptosis Induces Systemic Autoimmunity. Nat Immunol (2021) 22(9):1107–17. doi: 10.1038/s41590-021-00993-3 PMC8609402 34385713

[71] Juang YT Wang Y Solomou EE Li Y Mawrin C Tenbrock K. Systemic Lupus Erythematosus Serum IgG Increases CREM Binding to the IL-2 Promoter and Suppresses IL-2 Production Through CaMKIV. J Clin Invest (2005) 115(4):996–1005. doi: 10.1172/JCI22854 15841182 PMC1070410

[72] Kenny EF Herzig A Kruger R Muth A Mondal S Thompson PR. Diverse Stimuli Engage Different Neutrophil Extracellular Trap Pathways. Elife (2017) 6. doi: 10.7554/eLife.24437 PMC5496738 28574339

[73] Mao C Lei G Zhuang L Gan B. Ferroptosis as an Important Driver of Lupus. Protein Cell (2022) 13(5):313–5. doi: 10.1007/s13238-021-00892-1 PMC9008102 34826064

[74] Drakesmith H Nemeth E Ganz T. Ironing Out Ferroportin. Cell Metab (2015) 22(5):777–87. doi: 10.1016/j.cmet.2015.09.006 PMC4635047 26437604

[75] Gammella E Correnti M Cairo G Recalcati S. Iron Availability in Tissue Microenvironment: The Key Role of Ferroportin. Int J Mol Sci (2021) 22(6):ijms22062986. doi: 10.3390/ijms22062986 PMC7999357 33804198

[76] Wang CY Babitt JL. Hepcidin Regulation in the Anemia of Inflammation. Curr Opin Hematol (2016) 23(3):189–97. doi: 10.1097/MOH.0000000000000236 PMC4993159 26886082

[77] Wincup C Sawford N Rahman A. Pathological Mechanisms of Abnormal Iron Metabolism and Mitochondrial Dysfunction in Systemic Lupus Erythematosus. Expert Rev Clin Immunol (2021) 17(9):957–67. doi: 10.1080/1744666X.2021.1953981 PMC8452144 34263712

[78] Taysi S Gul M Sari RA Akcay F Bakan N. Serum Oxidant/Antioxidant Status of Patients With Systemic Lupus Erythematosus. Clin Chem Lab Med (2002) 40(7):684–8. doi: 10.1515/CCLM.2002.117 12241014

[79] Vanarsa K Ye Y Han J Xie C Mohan C Wu T. Inflammation Associated Anemia and Ferritin as Disease Markers in SLE. Arthritis Res Ther (2012) 14(4):R182. doi: 10.1186/ar4012 22871034 PMC3580577

[80] Phull AR Nasir B Haq IU Kim SJ. Oxidative Stress, Consequences and ROS Mediated Cellular Signaling in Rheumatoid Arthritis. Chemico-biol Interact (2018) 281:121–36. doi: 10.1016/j.cbi.2017.12.024 29258867

[81] Wu J Feng Z Chen L Li Y Bian H Geng J. TNF Antagonist Sensitizes Synovial Fibroblasts to Ferroptotic Cell Death in Collagen-Induced Arthritis Mouse Models. Nat Commun (2022) 13(1):676. doi: 10.1038/s41467-021-27948-4 35115492 PMC8813949

[82] Kalliolias GD Ivashkiv LB. TNF Biology, Pathogenic Mechanisms and Emerging Therapeutic Strategies. Nat Rev Rheumatol (2016) 12(1):49–62. doi: 10.1038/nrrheum.2015.169 26656660 PMC4809675

[83] Ling H Li M Yang C Sun S Zhang W Zhao L. Glycine Increased Ferroptosis *via* SAM-Mediated GPX4 Promoter Methylation in Rheumatoid Arthritis. Rheumatol (Oxford) (2022) pii:6524642. doi: 10.1093/rheumatology/keac069 35136972

[84] Katz WA Alexander S Bland JH Blechman W Bluhm GB Bonebrake RA. The Efficacy and Safety of Auranofin Compared to Placebo in Rheumatoid Arthritis. J Rheumatol Suppl (1982) 8:173–8. 6813481

[85] Celegato M Borghese C Casagrande N Mongiat M Kahle XU Paulitti A. Preclinical Activity of the Repurposed Drug Auranofin in Classical Hodgkin Lymphoma. Blood (2015) 126(11):1394–7. doi: 10.1182/blood-2015-07-660365 PMC4592277 26228484

[86] Yang L Wang H Yang X Wu Q An P Jin X. Auranofin Mitigates Systemic Iron Overload and Induces Ferroptosis *via* Distinct Mechanisms. Signal Transduct Target Ther (2020) 5(1):138. doi: 10.1038/s41392-020-00253-0 32732975 PMC7393508

[87] Xie Z Hou H Luo D An R Zhao Y Qiu C. ROS-Dependent Lipid Peroxidation and Reliant Antioxidant Ferroptosis-Suppressor-Protein 1 in Rheumatoid Arthritis: A Covert Clue for Potential Therapy. Inflammation (2021) 44(1):35–47. doi: 10.1007/s10753-020-01338-2 32920707

[88] Zhao T Yang Q Xi Y Xie Z Shen J Li Z. Ferroptosis in Rheumatoid Arthritis: A Potential Therapeutic Strategy. Front Immunol (2022) 13:779585. doi: 10.3389/fimmu.2022.779585 35185879 PMC8847160

[89] Gunther C Neumann H Neurath MF Becker C. Apoptosis, Necrosis and Necroptosis: Cell Death Regulation in the Intestinal Epithelium. Gut (2013) 62(7):1062–71. doi: 10.1136/gutjnl-2011-301364 22689519

[90] Erichsen K Milde AM Arslan G Helgeland L Gudbrandsen OA Ulvik RJ. Low-Dose Oral Ferrous Fumarate Aggravated Intestinal Inflammation in Rats With DSS-Induced Colitis. Inflamm Bowel Dis (2005) 11(8):744–8. doi: 10.1097/01.mib.0000174374.83601.86 16043990

[91] Carrier JC Aghdassi E Jeejeebhoy K Allard JP. Exacerbation of Dextran Sulfate Sodium-Induced Colitis by Dietary Iron Supplementation: Role of NF-Kappab. Int J Colorectal Dis (2006) 21(4):381–7. doi: 10.1007/s00384-005-0011-7 16133010

[92] Constante M Fragoso G Lupien-Meilleur J Calve A Santos MM. Iron Supplements Modulate Colon Microbiota Composition and Potentiate the Protective Effects of Probiotics in Dextran Sodium Sulfate-Induced Colitis. Inflamm Bowel Dis (2017) 23(5):753–66. doi: 10.1097/MIB.0000000000001089 28368910

[93] Biasi F Leonarduzzi G Oteiza PI Poli G. Inflammatory Bowel Disease: Mechanisms, Redox Considerations, and Therapeutic Targets. Antioxid Redox Signal (2013) 19(14):1711–47. doi: 10.1089/ars.2012.4530 PMC3809610 23305298

[94] Xu M Tao J Yang Y Tan S Liu H Jiang J. Ferroptosis Involves in Intestinal Epithelial Cell Death in Ulcerative Colitis. Cell Death Dis (2020) 11(2):86. doi: 10.1038/s41419-020-2299-1 32015337 PMC6997394

[95] Yin H Xu L Porter NA. Free Radical Lipid Peroxidation: Mechanisms and Analysis. Chem Rev (2011) 111(10):5944–72. doi: 10.1021/cr200084z 21861450

[96] Richard D Kefi K Barbe U Bausero P Visioli F. Polyunsaturated Fatty Acids as Antioxidants. Pharmacol Res (2008) 57(6):451–5. doi: 10.1016/j.phrs.2008.05.002 18583147

[97] Mayr L Grabherr F Schwarzler J Reitmeier I Sommer F Gehmacher T. Dietary Lipids Fuel GPX4-Restricted Enteritis Resembling Crohn’s Disease. Nat Commun (2020) 11(1):1775. doi: 10.1038/s41467-020-15646-6 32286299 PMC7156516

[98] Nishida T Miwa H Shigematsu A Yamamoto M Iida M Fujishima M. Increased Arachidonic Acid Composition of Phospholipids in Colonic Mucosa From Patients With Active Ulcerative Colitis. Gut (1987) 28(8):1002–7. doi: 10.1136/gut.28.8.1002 PMC1433134 3117625

[99] de Silva PS Olsen A Christensen J Schmidt EB Overvaad K Tjonneland A. An Association Between Dietary Arachidonic Acid, Measured in Adipose Tissue, and Ulcerative Colitis. Gastroenterology (2010) 139(6):1912–7. doi: 10.1053/j.gastro.2010.07.065 20950616

[100] Kagan VE Mao G Qu F Angeli JP Doll S Croix CS. Oxidized Arachidonic and Adrenic PEs Navigate Cells to Ferroptosis. Nat Chem Biol (2017) 13(1):81–90. doi: 10.1038/nchembio.2238 27842066 PMC5506843

[101] Jostins L Ripke S Weersma RK Duerr RH McGovern DP Hui KY. Host-Microbe Interactions Have Shaped the Genetic Architecture of Inflammatory Bowel Disease. Nature (2012) 491(7422):119–24. doi: 10.1038/nature11582 PMC3491803 23128233

[102] Bersuker K Hendricks JM Li Z Magtanong L Ford B Tang PH. The CoQ Oxidoreductase FSP1 Acts Parallel to GPX4 to Inhibit Ferroptosis. Nature (2019) 575(7784):688–92. doi: 10.1038/s41586-019-1705-2 PMC6883167 31634900

[103] Doll S Freitas FP Shah R Aldrovandi M da Silva MC Ingold I. FSP1 is a Glutathione-Independent Ferroptosis Suppressor. Nature (2019) 575(7784):693–8. doi: 10.1038/s41586-019-1707-0 31634899

[104] Brown CW Amante JJ Chhoy P Elaimy AL Liu H Zhu LJ. Prominin2 Drives Ferroptosis Resistance by Stimulating Iron Export. Dev Cell (2019) 51(5):575–86.e4. doi: 10.1016/j.devcel.2019.10.007 31735663 PMC8316835

[105] Gout PW Buckley AR Simms CR Bruchovsky N. Sulfasalazine, a Potent Suppressor of Lymphoma Growth by Inhibition of the X(C)- Cystine Transporter: A New Action for an Old Drug. Leukemia (2001) 15(10):1633–40. doi: 10.1038/sj.leu.2402238 11587223

[106] Tang B Zhu J Fang S Wang Y Vinothkumar R Li M. Pharmacological Inhibition of MELK Restricts Ferroptosis and the Inflammatory Response in Colitis and Colitis-Propelled Carcinogenesis. Free Radic Biol Med (2021) 172:312–29. doi: 10.1016/j.freeradbiomed.2021.06.012 34144192

[107] Iwamoto M Koji T Makiyama K Kobayashi N Nakane PK. Apoptosis of Crypt Epithelial Cells in Ulcerative Colitis. J Pathol (1996) 180(2):152–9. doi: 10.1002/(SICI)1096-9896(199610)180:2<152::AID-PATH649>3.0.CO;2-Y 8976873

[108] Di Sabatino A Ciccocioppo R Luinetti O Ricevuti L Morera R Cifone MG. Increased Enterocyte Apoptosis in Inflamed Areas of Crohn’s Disease. Dis Colon Rectum (2003) 46(11):1498–507. doi: 10.1007/s10350-004-6802-z 14605569

[109] Gunther C Martini E Wittkopf N Amann K Weigmann B Neumann H. Caspase-8 Regulates TNF-Alpha-Induced Epithelial Necroptosis and Terminal Ileitis. Nature (2011) 477(7364):335–9. doi: 10.1038/nature10400 PMC3373730 21921917

[110] Ma C Yang D Wang B Wu C Wu Y Li S. Gasdermin D in Macrophages Restrains Colitis by Controlling cGAS-Mediated Inflammation. Sci Adv (2020) 6(21):eaaz6717. doi: 10.1126/sciadv.aaz6717 32671214 PMC7314554

[111] Schwarzer R Jiao H Wachsmuth L Tresch A Pasparakis M. FADD and Caspase-8 Regulate Gut Homeostasis and Inflammation by Controlling MLKL- and GSDMD-Mediated Death of Intestinal Epithelial Cells. Immunity (2020) 52(6):978–93.e6. doi: 10.1016/j.immuni.2020.04.002 32362323

